# Fetal vascular malperfusion and other placental lesions in relation to maternal SARS-CoV-2 exposure: a case–control study

**DOI:** 10.3389/fmed.2026.1774613

**Published:** 2026-05-07

**Authors:** Irene Mwandoto, Marleen Temmerman, Ingrid Gichere, Patricia Okiro

**Affiliations:** 1 Department of Pathology, Aga Khan University Hospital, Nairobi, Kenya; 2 Center of Excellence in Women and Child Health, Nairobi, Kenya; 3 Department of Obstetrics and Gynaecology, Aga Khan University Hospital, Nairobi, Kenya

**Keywords:** coronavirus disease-19 (COVID-19), fetal vascular malperfusion (FVM), placenta, pregnancy, severe acute respiratory syndrome coronavirus 2 (SARS-CoV-2)

## Abstract

**Background:**

The morphologic characteristics of placentas associated with coronavirus disease-19 (COVID-19) have been described but with varying findings. Studies conducted at the onset of the pandemic reported frequency of Fetal Vascular Malperfusion (FVM), an umbrella term that describes occlusive vasculopathies that affect fetal circulation and their associated sequelae. Morphologic manifestations include thrombosis, avascular villi and vascular stromal karyorrhexis. Associated outcomes include fetal growth restriction, thromboembolic events such as neonatal stroke and fetal demise. Placental lesions associated with a severe acute respiratory syndrome-related coronavirus 2 (SARS-CoV-2) infection present a unique picture in histology, especially in the attempt to decipher the underlying pathophysiology of the disease. This study aimed to determine whether there was an association between maternal SARS-CoV-2 infection and placental findings of FVM and to describe the prevalence of other histopathological changes in placentas from COVID-19 exposed pregnant women.

**Materials and methods:**

Two hundred thirty-four (234) placentas, including 33 cases with features of FVM and 201 controls without FVM, were collected from women delivering in Aga Khan University Hospital, Nairobi, recruited between June 2021 and November 2022. Histological evaluation, as per the Amsterdam placental workshop group consensus, was performed. To determine the association, a univariate analysis was conducted using a Fisher’s Exact test for categorical data.

**Results:**

Fetal vascular malperfusion was observed in 14.1% (*n* = 33) of placentas. Of these, 45.5% (*n* = 15) were from SARS-CoV-2 PCR-positive mothers, whereas 54.5% (*n* = 18) were PCR-negative. Placentas from 201 participants did not show features of FVM. Of these, 59 (29.4%) placentas were from PCR/antigen SARS-CoV-2 positive women, while 142 (70.6%) were from those who were PCR/antigen SARS-CoV-2 negative. No statistically significant association was observed between exposure to COVID-19 during pregnancy and FVM [OR = 2.01 (C1 95%:4.24); *p* = 0.069].

**Conclusion:**

No statistically significant association between maternal SARS-CoV-2 infection during pregnancy and FVM was observed, however an association cannot be definitively excluded. Chorangiosis was frequently observed, suggesting possible hypoxia-related placental changes. Larger studies are needed to determine whether SARS-CoV-2 exposure contributes to these lesions.

## Introduction

The coronavirus disease 2019 (COVID-19), whose etiology is severe acute respiratory syndrome coronavirus 2 (SARS-CoV-2), following global dissemination, was declared a pandemic by the World Health Organization (WHO) in March 2020 (1). It has since then resulted in significant morbidity and mortality, with different virulent strains emerging during its evolution (2), necessitating the need for urgent vaccine development and effective treatment strategies. While infection has since waned, its effects have been documented, particularly in populations that were deemed vulnerable, such as pregnant women.

Maternal exposure to the virus has shown evidence of increased risk for unfavorable outcomes, including miscarriage (3), preterm delivery (4, 5), fetal growth restriction (4), stillbirth and even the likelihood of vertical transmission (6), highlighting a need for placental examination in women exposed to SARS-CoV-2 during pregnancy.

Since the onset of the pandemic, histopathologic evaluation of placentas collected from women infected during pregnancy has yielded a variability of morphologic outcomes with no apparent correlates (7, 8). Initial studies were mainly case studies and case reviews with limited numbers (6). Disease progression saw the attempt to include larger cohorts with the incorporation of controls. Lesions described include fetal vascular malperfusion (FVM), inflammatory features and maternal vascular malperfusion (MVM) (7, 9, 10). However, the full extent and nature of these abnormalities remain insufficiently explored. Each lesion has been described with variable frequency, occurring independently or overlapping.

Capitalizing on the placenta as a diagnostic and prognostic tool is of emerging interest, as this equips those in the clinical set-up with evidence-based management strategies (11). With the emergence of COVID-19 as a denominator of unfavorable maternal and fetal outcomes, it becomes imperative to investigate any associations between exposure to the virus during pregnancy and the development of placental lesions. Entities such as FVM are of interest not only due to limited perinatal diagnostic modalities available to accurately predict the presence of FVM in utero (12) but also because of the possibility of it being a consequence of the pro-thrombotic effects of COVID-19 (13). The desire to establish the presence of FVM and elucidate whether it would be associated with maternal SARS-CoV-2 infection would be rooted in the need to not only inform follow-up of neonates born to these mothers with a history of COVID-19 infection during pregnancy but also contribute to a deeper understanding of COVID-19’s impact on pregnancy.

## Literature review

### Placenta: anatomy

The placenta is an essential organ of pregnancy that supports fetal development in utero. Its functions include facilitating nutrient transfer between mother and fetus, producing hormones such as progesterone and estrogen that support pregnancy, eliminating waste and forming a selective barrier to maintain a favorable microenvironment and for fetal protection (14).

Placentation begins when the trophectoderm of the blastocyst (pre-implantation embryo) differentiates into trophoblasts, which subsequently differentiate into extra villous trophoblastic cells that invade the maternal decidua to establish maternal-fetal circulation. The blastocyst’s underlying extraembryonic mesoderm gives rise to a fibrovascular network and population of Hofbauer cells (14). The fetal elements arise from the chorionic sac while the maternal components are derived from the decidualized endometrium, with an intervillous space in between filled with maternal blood (1). Within the intervillous space is a branched villous tree that arises from a stem villus that attaches to the basal plate (anchoring villi) and continuously differentiates with increasing gestational age so that beyond 20 weeks terminal villi are formed primarily (14).

Fetal syncytiotrophoblasts cells form an avenue of communication between the intervillous space where maternal blood flows and the fetus. It is through this middle ground that exposure to circulating insults present in maternal blood may occur. Syncytiotrophoblasts, however, lack intercellular junctions and receptors, thereby forming a barrier that restricts pathogenic entry and remains resistant to inflammation-induced modulation (1, 15).

The integrity of the placenta is reliant on both the structural and immunomodulatory elements in place. Structurally, the syncytium forms a barrier between the mother and fetus while regulation of nuclear factor-kB, interferon signaling and autophagocytotic activity triggered by micro-ribonucleic acid (m-RNA) serve as an immune defense against pathogens (1). Circumvention or overburdening of these homeostatic mechanisms by pathogens may result in in-utero or transplacental infection with consequent adverse outcomes (16).

Mature placental gross morphology is characterized by an oval to circular disc ranging in size from 15 to 20 cm in diameter (median 22 cm), 2.5 cm in thickness with an average weight of 500 grams at term (14, 17). The disc is composed of a chorionic plate (fetal surface) and a basal plate (maternal surface). The umbilical cord inserts centrally on the fetal surface with an average length of 55 cm (50–60 cm) and diameter of 0.8 to 2 cm (18). Studies have shown that marked deviations in placental morphology and morphometry are useful clues of adverse pregnancy outcomes (19–21).

### Placental morphology in viral infections

Viral infections have been shown to significantly affect pregnancy outcomes (22). Histopathological assessment of placentas in these scenarios has offered insight into the impact and possible histological outcomes of infection during pregnancy (23). Placental injury is attributed to downregulation of immune defense systems, immune evasion strategies, collateral damage in the face of mounted inflammatory response or via receptor-mediated trophoblastic infection (23).

Microscopic findings associated with transmissible viruses such as human immunodeficiency virus (HIV), cytomegalovirus (CMV) and rubella include immature distal villi and diminished vasculature in HIV, delayed villous maturation, chronic villitis and occasional eosinophilic intranuclear inclusions in CMV and intervillositis, trophoblastic necrosis, umbilical vasculitis in rubella (23).

Previous epidemics involving coronaviridae, Middle East respiratory syndrome coronavirus (MERS-CoV-2) and severe acute respiratory syndrome (SARS), offered limited input on the pathophysiologic characteristics of placentas. One study that examined seven placentas with history of SARS during pregnancy showed two with features in keeping with FVM, evidenced by the presence of avascular villi and intraluminal thrombi (24).

### Placental histology in COVID-19

Severe acute respiratory syndrome coronavirus 2, a single-stranded RNA virus, requires the presence of receptors such a s angiotensin-converting enzyme 2 (ACE-2) or Dipeptidyl peptidase 4 to facilitate entry into the host cell and priming proteases including Transmembrane protease serine 2 (TMPRSS2), Trypsin, Furin or Cathepsin L that make the viral spike (S) protein fully functional for infectivity to prevail (1).

Angiotensin-converting enzyme 2 receptors, Furin and Trypsin have been shown to be present on cytotrophoblasts and syncytiotrophoblasts that line chorionic villi, thus providing a possible avenue for viral entry and consequent infection. Viral entry and multiplication trigger an immune-mediated reaction that may result in placental injury, transplacental transmission or both (23).

### FVM and MVM in SARS-CoV-2

Fetal vascular malperfusion is an umbrella term that describes the downstream effects of occlusive vasculopathy in the fetal circulation pathway (25). The risk of developing FVM is influenced by maternal factors such as hypercoagulable states like thrombophilia and fetal factors such as hepatic disease. Adverse effects of FVM include fetal growth restriction, thromboembolic events such as neonatal stroke with consequent neurodevelopmental challenges (12).

Gross features that may be indicative of this lesion include umbilical cord hypo- or hyper coiling, abnormal cord insertion or umbilical cord knots which may interfere with laminar flow of blood with resultant thrombus formation (26). Microscopic manifestations of occlusive effects are seen as avascular chorionic villi, villous stromal karyorrhexis or intraluminal thrombi. These lesions are grades based on the extent of involved villi or presence and number of intraluminal thrombi into low and high-grade (27). The reported incidence of FVM ranges from 5% in term live births to 19% in fetal deaths (28).

FVM was a frequent finding in studies conducted at the onset of the pandemic (7, 29, 30). The postulated pro-inflammatory, pro-coagulant effects of viral infection are thought to support the pathogenic basis of FVM (31). Increased expression of von Willebrand factor (vWf), a procoagulant factor was noted in the endothelia of the decidua and chorionic villi of women with symptomatic COVID-19. On microscopy, intraluminal thrombi were seen, along with parenchymal infarcts and intervillositis (32). Whether there was an association between timing of maternal SARS-CoV-2 infection and development of FVM was explored in a prospective cohort study that found the presence of FVM to be higher (53.8%) in women with PCR-positive SARS-CoV-2 infection within 2 weeks of delivery compared with those in the non-acute setting (18.8%) and PCR-negative controls (13.2%) (*p* < 0.001) (33).

Maternal vascular malperfusion on the other hand denotes a pattern of lesions that result due to impaired maternal blood flow (34). It is characterized by decidual arteriopathy (atherosis, fibrinoid necrosis, arterial thrombosis and mural hypertrophy), infarcts, accelerated villous maturation and distal villous hypoplasia on microscopy (1). MVM has been shown to be associated with maternal obesity, diabetes and hypertensive disorders (35). Adverse fetal outcomes include fetal growth restriction, oligohydramnios, preterm birth and fetal demise (34).

The presence of MVM in association with SARS-CoV-2 infection during pregnancy has also been documented (10, 36). Despite differing etiologies, both malperfusion lesions have been linked to fetal growth restriction, a complication associated with unfavorable neurodevelopmental outcome (37).

## Materials and methods

### Study design and case definition

A case–control study nested in a WHO parent study entitled ‘*A prospective cohort study investigating maternal, pregnancy and neonatal outcomes for women and neonates infected with SARS-CoV-2’* was designed. This was an unmatched case–control study, as no overall significant differences or confounders were noted in the participants, and cases were determined post-microscopic analysis.

Cases were defined by placentas exhibiting changes in keeping with FVM, including thrombosis, avascular villi and vascular stromal karyorrhexis. Placentas without the above-described changes were assigned to the control arm.

### Study setting and population

The study was conducted at the Aga Khan University Hospital, Nairobi, Pathology Department, in collaboration with the Department of Obstetrics and Gynecology.

The recruitment of pregnant women into the parent study commenced in June 2021.

The study participants were pregnant women in the antenatal clinics and labor ward in AKUHN who were recruited and grouped based on reverse transcriptase polymerase chain reaction (RT-PCR), antigen, serology status and COVID-19 vaccination status. Testing was performed at the point of admission as per COVID-19 protocol instituted at the onset of the pandemic or at the time of recruitment when attending antenatal clinic.

Testing was done with nasopharyngeal swabs using Allplex™ SARS-CoV-2 Assay, Seegene/Realstar® rRT-PCR detection kit by Altona Diagnostics which amplified a SARS-CoV-2 specific sequence of the S gene. Nasopharyngeal swabs were placed in viral transport medium, nucleic acids were then isolated and purified by an automated extraction system. Extraction of RNA was performed as per the manufacturer’s instructions.

Alternatively, antigen testing was also performed for those without a prior PCR result during ANC enrollment, with nasopharyngeal swabs using Panbio™ COVID-19 Ag Rapid test device by Abbott, a lateral flow immunoassay that detects the nucleocapsid antigen of the SARS-CoV-2 virus. For serology testing, 4 milliliters (mL) of blood was collected aseptically from the antecubital vein and centrifuged for 15 min at 1000 rotations per minute to extract serum. Anti-SARS-CoV-2 antibodies were estimated in the serum sample by Enzyme-linked Immunosorbent Assay (ELISA) using two assays. Wantai SARS-CoV-2 Ab to detect total anti-spike antibodies (IgG and IgM), which are produced following natural infection or vaccination. Platelia SARS-CoV-2 Total Ab assay by BIO-RAD was used to detect total anti-nucleocapsid (anti-NCP) antibodies (IgM, IgA and IgG), which are generated only after natural infection.

The parent study defined exposure status as a positive RT-PCR (Allplex™ SARS-CoV-2 Assay, Seegene/Realstar® SARS-CoV-2 RT-PCR Kit 1.0, Altona Diagnostics), Antigen (Panbio™ COVID-19 Ag Rapid Test Device, Abbott) or serology (Wantai SARS-CoV-2 Ab Elisa for unvaccinated/ Platelia SARS-CoV-2 Total Ab, BIO-RAD), during pregnancy and until 48 h following delivery. Those with negative serology and RT-PCR/antigen status were placed in the unexposed arm.

The present study defined exposure status as testing positive for COVID-19 by RT-PCR or antigen during the current pregnancy indicating an acute infection during the gestational period. Serological testing performed demonstrated prior infection but did not inform on timing of infection, antibody positivity was therefore not used to define exposure during pregnancy. These participants also had their placentas collected and examined. This approach minimized potential misclassification of infection timing. Written informed consent for histology was sought from all these participants.

The study population comprised all placentas collected from women recruited consecutively during the parent study period (June 2021 to November 2022). Pregnant women aged 18 years and above delivering at AKUHN were included in the study. Maternal comorbidities were defined as any diagnosed medical condition during pregnancy. Only diabetes and hypertension were included in statistical analyses due to clinical relevance and sufficient sample size. Other rare comorbidities such as sickle cell disease, fibromyalgia, and thrombocytopenia were recorded but not analyzed due to low frequency and heterogeneity. Women with conditions known to predispose to FVM such as thrombophilia and SLE were excluded.

### Sampling

**Figure 1:**
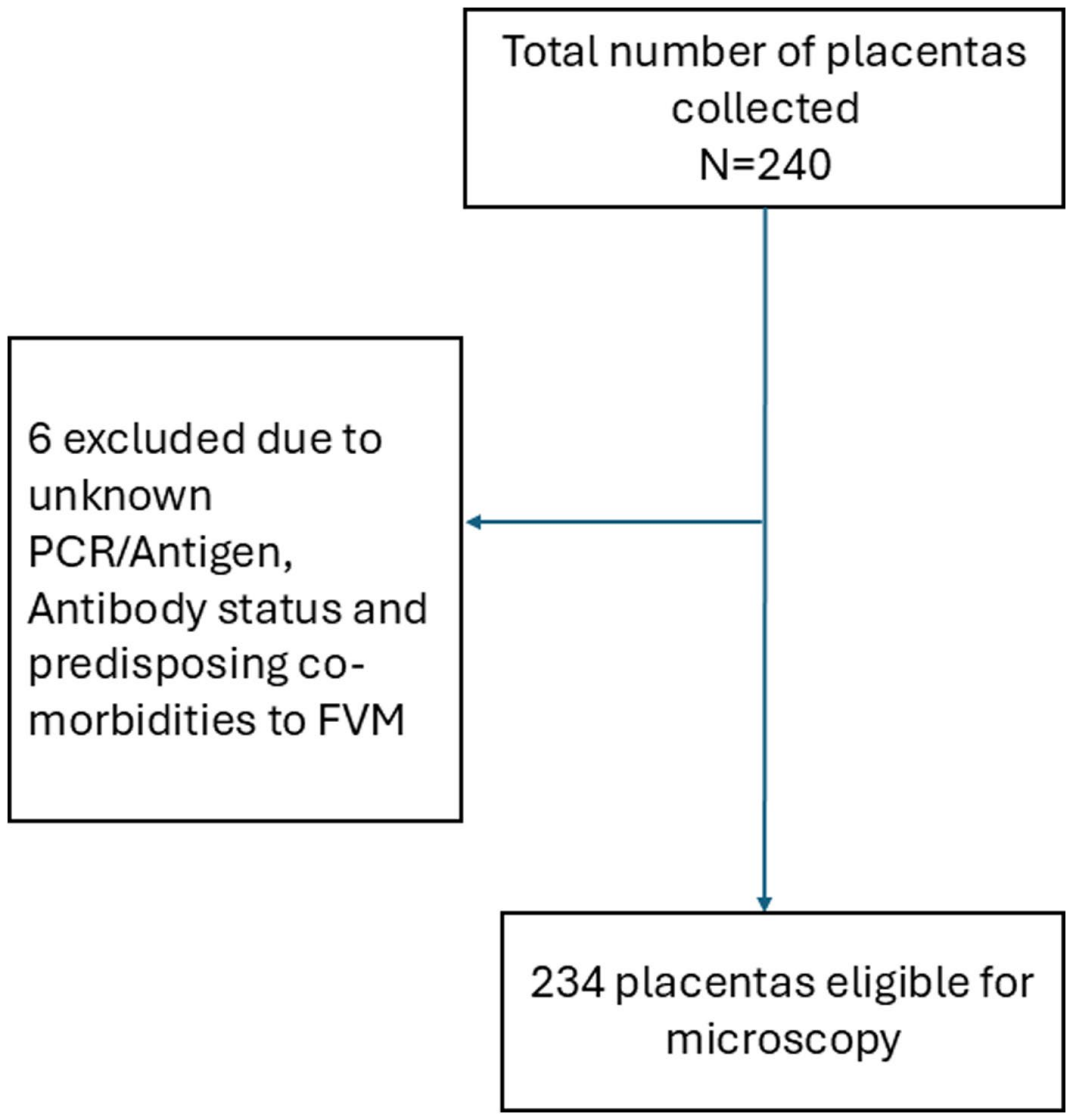
Flow chart of placentas included in microscopic examination.

A census sampling approach was adopted, precluding the calculation of sample size. Placentas collected from women recruited within the parent study period informed the sample size. All eligible placentas from women who provided consent were collected and examined. A total of 240 placentas were collected. Six were excluded due to unknown PCR/antigen, antibody status (*n* = 5) and predisposing co-morbidities to FVM (*n* = 1). A total of 234 placentas met the inclusion criteria and were included in the final analysis (Figure 1). For analysis, placentas from PCR/antigen positive participants (*n* = 74) and serology positive participants (*n* = 212) were each examined separately to provide focused assessments of histopathological changes associated with confirmed or prior SARS-CoV-2 exposure.

### Sample collection and laboratory procedures

Following delivery, placentas from recruited women were collected and placed in 10% neutral buffered formalin for fixation. Gross features, including placental weight, membrane, umbilical cord and parenchymal features, were evaluated using a standardized grossing template.

Sectioning was done at a one-centimeter interval and examined for any macroscopic lesions. Representative sections submitted included two of the umbilical cord-fetal and maternal ends, two of the membrane rolls, three full-thickness parenchyma sections and additional sampling of any lesions present.

The sections underwent routine processing and paraffin embedding (Sakura Tissue-Tek® VIP™ 5 Jr., Japan) to prepare tissue blocks. Blocks were sectioned at four microns on a microtome (Leica RM2125RTS, Nussloch, Germany), mounted on slides, deparaffinized and rehydrated through graded alcohols. Subsequent staining with hematoxylin was done to highlight cell nuclei, followed by rinsing and differentiation. Eosin was subsequently applied to stain the cytoplasm. The slides were then dehydrated through graded alcohols, cleared with xylene and cover slipped for light microscopy evaluation (Dako CoverStainer).

Microscopic evaluation was performed based on the Amsterdam Placental Workshop Group Consensus Statement (38).

First, the investigator examined all the slides alone under an Olympus CX31 microscope (model CX31RTSF, Japan) using both low (x4, x10) and high power (x20, x40) to examine the entire tissue mounted on the slide and then jointly with an experienced perinatal and pediatric pathologist with an Olympus (model U-SDO3, Japan) two-header microscope. Both were partially blinded to the participants’ PCR/antigen and antibody status.

Sections were examined for FVM, any one feature of the following microscopic findings were concluded as FVM: avascular villi, intraluminal thrombi, villous stromal karyorrhexis. Grading was as follows: Low-grade: <5 villi per focus of avascular or karyorrhectic villi, High-grade: >45 avascular villi over 3 sections examined or >15 avascular villi per section with or without thrombi or 2 or more occlusive or nonocclusive thrombi in major stem villi or chorionic plate. MVM was defined by any one finding of decidual arteriopathy (acute atherosis, mural hypertrophy, fibrinoid necrosis, arterial thrombosis and persistence of intramural endovascular trophoblasts), infarcts, accelerated villous maturation or distal villous hypoplasia. Acute inflammation (chorioamnionitis) was diagnosed based on the presence of neutrophils in the chorion with extension to amnion. Chronic inflammation was assessed in the villi, intervillous space and decidua, characterized by the presence of lymphocytes, histiocytes and plasma cells, respectively. Additional findings not included in the Amsterdam criteria such as amniocyte hyperplasia, fibrin deposition, chorangiosis and pigmented macrophages were documented to provide a comprehensive assessment of placental pathology. These features were analyzed descriptively and were not included with malperfusion outcomes in statistical comparisons. The finding of chorangiosis was based on Altshuler’s criteria (>10 capillaries in 10 terminal chorionic villi in 3 ×10 power fields) (39).

### Statistical analysis

Each sample’s clinical, demographic characteristics and histological data were entered into a Microsoft Excel 2016 database. The results for microscopic placental lesions were recorded in a binary format (absent or present for both cases and controls). Categorical data were presented as frequencies and percentages, and continuous data as means and standard deviation. Univariate analyses used Fisher’s Exact Test to determine group association for categorical data. *p* values of < 0.05 were considered statistically significant.

### Ethical considerations

Ethical approval was sought and received from the Aga Khan University Ethical Review Committee (2021/IERC-163 (v4)). The included participants were nested under a parent study whose ethical approval had been granted by the Aga University Ethical Review Committee under study number 2020/IERC-153 (v2). Written informed consent for histology was obtained from all participants. All patient data was de-identified and stored in a password-protected computer accessible only to the primary investigator.

## Results

### Baseline clinical characteristics of all participants

Maternal age at delivery averaged 32.9 years, with a median of 33 years and an age range of 21–47 years. The mean gestation age of delivery was 38.6 weeks, ranging from 32 to 42 weeks. Comorbidities were present in 21.8% (51/234), with diabetes seen in 25 of the participants (21 with gestational diabetes and 4 with type 2 diabetes) and hypertension in 14 participants. The remaining 12 participants had other comorbidities including sickle cell disease, fibromyalgia, and hypothyroidism, which were not analyzed due to low frequency and heterogeneity.

Of 234 placentas, 31.6% (74/234) were from women with a positive PCR/antigen SARS-CoV-2 status during pregnancy. Serological testing was performed on 212 participants, with 75% (159/212) showing antibody positivity (Table 1).

**Table 1:** Baseline clinical and gross placental characteristics of all participants.

Clinical and gross placental characteristics		*N* = 234	
		N/Mean(SD)	
Age (years)		32.9 [4.9]	
Gestational age (weeks)		38.6 [1.5]	
PCR/antigen Status	Negative	160	68.4%
	Positive	74	31.6%
Antibody done	No	22	9.4%
	Yes	212	90.6%
Antibody Status	Negative	53	25.0%
	Positive	159	75.0%
Comorbidities	Hypertension	14	27.5%
	Diabetes	25	49.0%
Placenta weight (g)		481.5 [94.8]	
Umbilical Insertion	Central	54	23.1%
	Eccentric	158	67.5%
	Marginal	22	9.4%
	Velamentous	5	2.1%
Parenchyma	Normal	190	81.2%
	Not normal	44	18.8%

### Gross placental characteristics

**Figure 2:**
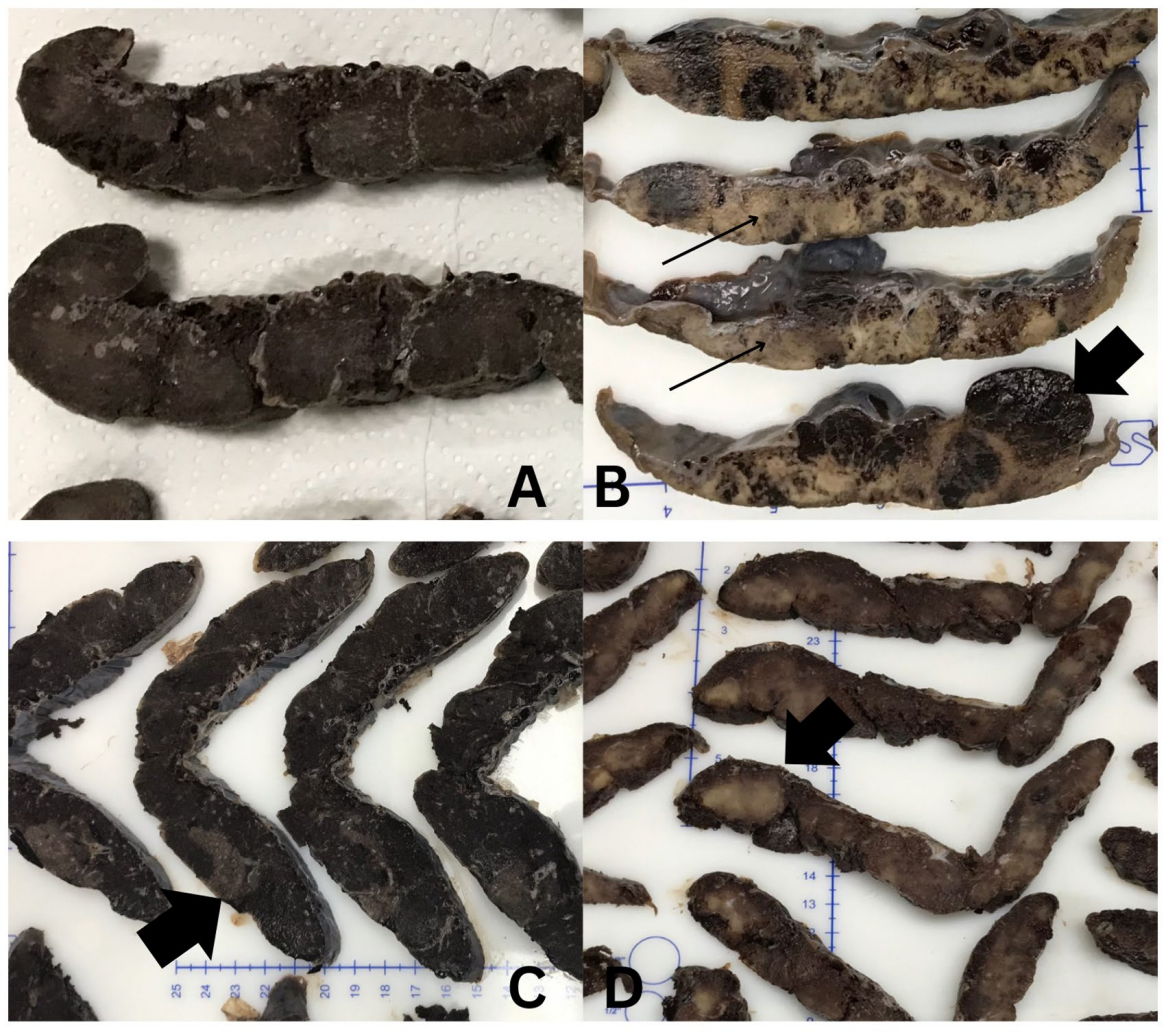
A selection of gross parenchymal findings in women with COVID-19: **(A)** Normal tan-brown parenchyma. **(B)** Tan-white areas (black arrows), hemorrhagic areas (block arrow) microscopy shown in Figure 5D. **(C)** Pale parabasal areas (block arrow)microscoy shown in Figure 5A. **(D)** Peripheral infarct (block arrow).

Morphologic examination was performed for 234 placentas. The mean placental weight was 481.5 grams, and most placentas showed an eccentric umbilical cord insertion site (67.5%) (158/234). The macroscopic parenchymal appearance of 81.2% (190/234) placentas was normal, with 18.8% (44/234) exhibiting gross aberrations (Table 1). As depicted in Figure 2, those with macroscopically visible abnormalities showed a spectrum of foci, the majority being peripheral infarcts (22.7%, 10/44), others showing pale patchy areas (Figure 2C) (6.8%, 3/44), one with multiple hemorrhagic foci (2.3%, 1/44) (Table 2).

**Table 2:** Abnormal gross parenchymal findings.

Macroscopic parenchymal findings	Number (*n* = 44)	Proportion (%)
Basal pale foci	1	2.3%
Central haematoma	1	2.3%
Central infarct	1	2.3%
Central infarct, peripheral haemorrhagic focus	1	2.3%
Haemorrhagic focus	1	2.3%
Pale cut surface	1	2.3%
Pale parabasal focus	1	2.3%
Pale patchy areas	6	13.6%
Parabasal tan-white focus	1	2.3%
Patchy pale areas	3	6.8%
Peripheral haematoma	2	4.5%
Peripheral infarct	10	22.7%
Peripheral parabasal tan-white focus	1	2.3%
Pseudocyst	4	9.1%
Pseudocyst and parabasal tan-white focus	1	2.3%
Solid central tan-brown focus, marginal retroplacental clot	1	2.3%
Solid tan-white foci	1	2.3%
Subchorionic haematoma	1	2.3%
Subchorionic tan-white foci	2	4.5%
Tan white with multiple hemorrhagic foci	1	2.3%
Tan-brown focus	1	2.3%
Tan-white foci	2	4.5%

### Microscopic placental characteristics

Histological features identified in all 234 examined placentas were stratified as per the patterns of injury described in the Amsterdam Placental Workshop Group Consensus recommendations (Table 3). Fifty placentas (21.4%) were within normal limits. Fetal vascular malperfusion was present in 14.1% (33/234) of placentas. At least one feature of MVM was seen in 4.7% (11/234). Acute inflammatory features were identified in 9.4% (22/234), while chronic inflammation was present in 12.4% (12/234). Additional findings across all placentas included amniocyte hyperplasia, chorangioma, chorangiomatosis, chorangiosis, delayed villous maturation, excessive fibrin, focal hydropic change, hemorrhagic infarct intervillous hematoma, laminar necrosis, peripheral infarcts, pigmented macrophages, retroplacental hematoma and subchorionic hematoma. These findings were present in 66.2% (*n* = 155) of examined placentas. Among them, chorangiosis was the most frequent, observed in 32.9% (*n* = 77) of placentas.

**Table 3:** Summary of microscopic findings seen in all placentas.

Microscopic findings		Number (*n* = 234)	Proportion (%)
Normal findings	No	184	78.6%
	Yes	50	21.4%
MVM	Absent	223	95.3%
	Present	11	4.7%
FVM	Absent	201	85.9%
	Present	33	14.1%
Acute inflammation	Absent	212	90.6%
	Present	22	9.4%
Chronic inflammation	Absent	205	87.6%
	Present	29	12.4%
Other findings	Absent	79	33.8%
	Present	155	66.2%
Other findings present	Amniocyte hyperplasia	30	12.8%
	Chorangioma	3	1.3%
	Chorangiomatosis	3	1.3%
	Chorangiosis	77	32.9%
	Delayed maturation	28	12.0%
	Excessive fibrin	1	0.4%
	Focal hydropic change	1	0.4%
	Haemorrhagic infarct	1	0.4%
	Intervillous haematoma	27	11.5%
	Laminar necrosis	4	1.7%
	Peripheral infarct	13	5.6%
	Pigmented macrophages	9	3.8%
	Retroplacental haematoma	7	3.0%
	Subchorionic haematoma	14	6.0%

### Clinical and gross placental characteristics of cases and controls

**Figure 3:**
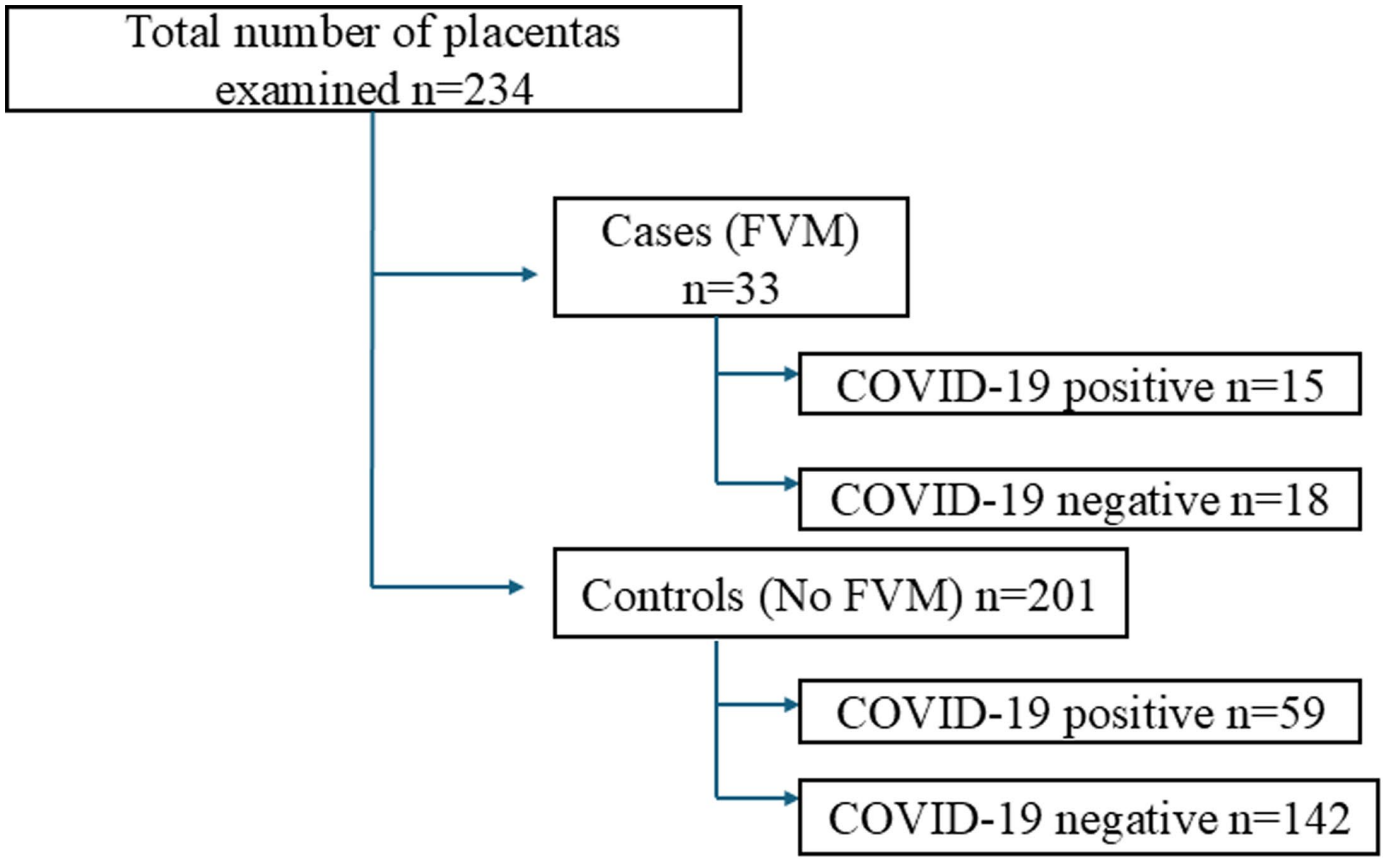
Flow diagram showing distribution of cases and controls by SARS-CoV-2 status.

Both cases and controls showed a similar distribution, with a mean age of 31.6 years in cases and 33.1 years in controls. Gestational age averaged 38.7 weeks in cases and 38.6 weeks in controls. Cases were PCR/antigen positive in 45.5% (15/33), while controls lacking FVM features had 29.4% (59/201) positivity. These distributions are illustrated in Figure 3. Placental weight averaged 484.5 g in cases and 481.1 g in controls, with umbilical insertion being eccentric at 72.7% (24/33) and 66.7% (134/201) in the control arm. Cases that demonstrated gross parenchymal findings were 18.2% (6/33), while controls had 18.9% (38/201) with macroscopic anomalies (Table 4).

**Table 4:** Clinical and gross placental characteristics of cases (*n* = 33) and controls (*n* = 201).

Clinical and gross characteristics		Case		Control	
		*n* = 33 *N*/Mean(SD)		*n* = 201 *N*/Mean (SD)	
Age (years)		31.6 [5.0]		33.1 [4.8]	
Gestational age (weeks)		38.7 [1.6]		38.6 [1.5]	
Parity	0–2	26	78.8%	164	81.6%
	≥ 3	7	21.2%	37	18.4%
Losses	0–2	32	97.0%	197	98.0%
	≥ 3	1	3.0%	4	2.0%
PCR/antigen status	Negative	18	54.5%	142	70.6%
	Positive	15	45.5%	59	29.4%
Antibody done	No	5	15.2%	17	8.5%
	Yes	28	84.8%	184	91.5%
Antibody status	Negative	7	21.2%	46	22.9%
	Positive	21	63.6%	138	68.7%
Comorbidities	Yes	4	12.1%	47	23.4%
	No	29	87.9%	154	76.6%
Comorbidities	Hypertension	2	50.0%	12	25.5%
	Diabetes	2	50.0%	23	48.9%
Placenta weight (g)		484.5 [110.6]		481.1 [92.3]	
Umbilical insertion	Central	6	18.2%	48	23.9%
	Eccentric	24	72.7%	134	66.7%
	Marginal	3	9.1%	19	9.5%
	Velamentous	0	0.0%	5	2.5%
Parenchyma	Normal	27	81.8%	163	81.1%
	Not Normal	6	18.2%	38	18.9%

### Association of SARS-CoV-2 and placental FVM

**Figure 4:**
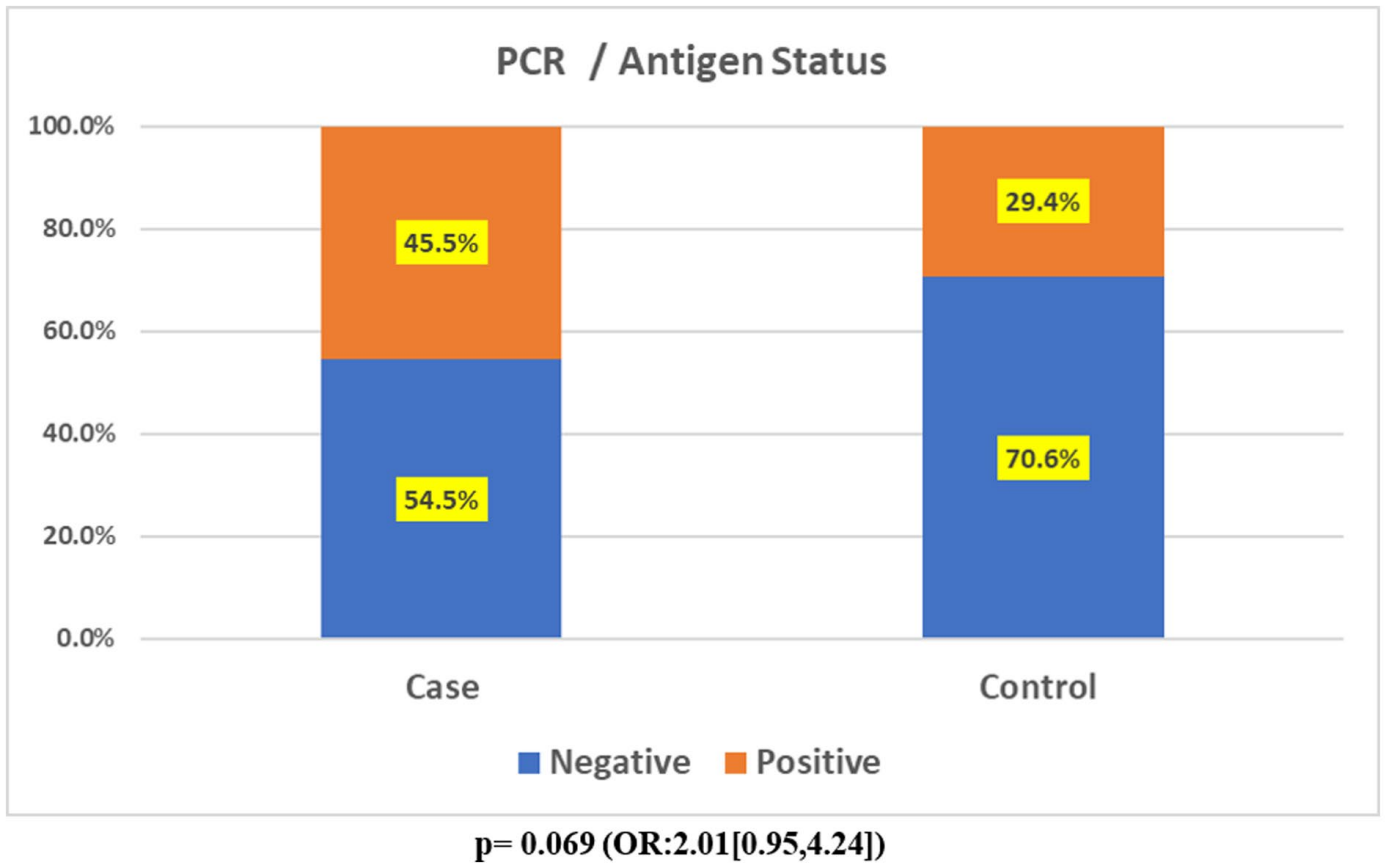
Stacked charts showing association of PCR/antigen status with cases (FVM) and controls (No FVM).

Maternal SARS-CoV-2 infection was present in 45.5% of cases compared to 29.4% (59/201) of controls. Although SARS-CoV-2 infection was associated with higher odds of FVM (OR 2.01, 95%CI 0.95–4.24), this association did not reach statistical significance (*p* = 0.069; Figure 4).

### Proportion of FVM grades in PCR/antigen-negative and positive participants

Among the cases with FVM, distribution of low- and high-grade lesions was similar between SARS-CoV-2 positive and negative participants (Table 5). Low-grade FVM was observed in 66.7% (10/15) of SARS-CoV-2 positive participants and 66.7% (12/18) of SARS-CoV-2 negative participants while high-grade FVM was seen in 33.3% (5/15) and 27.8% (5/18) respectively.

**Table 5:** FVM grade and PCR/antigen status.

FVM grade		PCR/antigen status				*p* value
		Negative (*n* = 18)		Positive (*n* = 15)		
FVM grade	High	6	33.3%	5	33.3%	1.000
	Low	12	66.7%	10	66.7%	

### Prevalence of placental lesions in COVID-19-positive participants

Of the 234 placentas included in the study, 74 were collected from PCR/antigen positive participants, including both cases and controls. Maternal vascular malperfusion was seen in 9.5% (7/74), acute inflammation in 10.8% (8/74) and chronic inflammation in 18.9% (14/74). Delayed villous maturation was observed in 16.07% (9/74), while amniocyte hyperplasia was present in 10.71% (6/74). Chorangiomatosis and chorangioma were rare, each identified in 1.4% (1/74) of placentas (Table 6).

**Table 6:** Summary of microscopic findings in placentas from PCR/antigen positive women.

Microscopic findings		PCR/antigen positive (*n* = 74)	
		Number	Proportion (%)
MVM	Absent	67	90.50%
	Present	7	9.50%
FVM	Absent	59	79.70%
	Present	15	20.30%
Acute inflammation	Absent	66	89.20%
	Present	8	10.80%
Chronic inflammation	Absent	60	81.10%
	Present	14	18.90%
Other findings present	Amniocyte hyperplasia	6	10.71%
	Chorangioma	1	1.79%
	Chorangiomatosis	1	1.79%
	Chorangiosis	27	48.21%
	Delayed maturation	9	16.07%
	Excessive fibrin	0	0.00%
	Focal hydropic change	0	0.00%
	Haemorrhagic infarct	1	1.79%
	Intervillous haematoma	8	14.29%
	Laminar necrosis	2	3.57%
	Peripheral infarct	5	8.93%
	Pigmented macrophages	2	3.57%
	Retroplacental haematoma	0	0.00%
	Subchorionic haematoma	4	7.14%

### Microscopic findings in serology-positive participants

**Figure 5:**
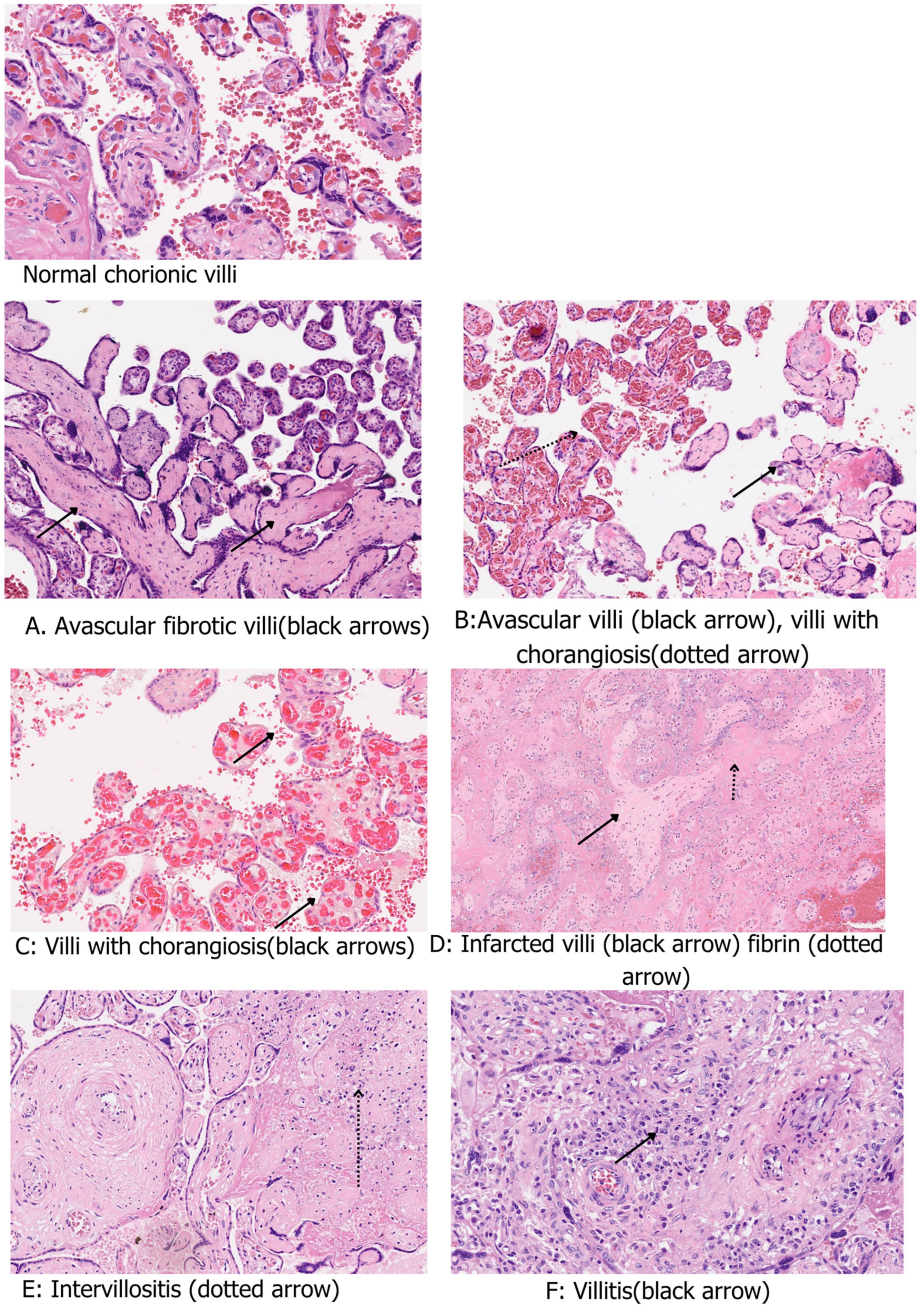
Representative placental micrographs from women with COVID-19 during pregnancy (H&E, X20) showing **(A)** Avascular fibrotic chorionic villi (black arrows). **(B)** Avascular villi (black arrow) with chorangiosis (dotted arrow). **(C)** Chorangiosis (black arrows). **(D)** Infarcted villi (black arrow) and perivillous fibrin (dotted arrow). **(E)** Intervillositis (dotted arrow). **(F)** Villitis (black arrow). Top left micrograph shows normal chorionic villi.

Among participants who underwent serology testing (*n* = 212), 75% (159/212) were antibody positive. Of these antibody positive women, 56.1% (119/212) tested negative for PCR/antigen at enrollment. Placentas from the antibody positive cohort demonstrated FVM (Figure 5B) in 10.1% (12/212) and MVM in 1.7% (2/212). Acute inflammation was observed in 6.7% (8/212), chronic inflammation (Figures 5E,F) in 9.2% (11/212), and chorangiosis in 49.4% (40/212) (Figures 5B,C; Table 7).

**Table 7:** Microscopic findings according to PCR/antigen and antibody status.

Microscopic findings		PCR/antigen status							
		Negative				Positive			
		Antibody status							
		Negative		Positive		Negative		Positive	
FVM	Absent	33	86.80%	107	89.90%	13	86.70%	31	77.50%
	Present	5	13.20%	12	10.10%	2	13.30%	9	22.50%
MVM	Absent	36	94.70%	117	98.30%	15	100.00%	34	85.00%
	Present	2	5.30%	2	1.70%	0	0.00%	6	15.00%
Acute inflammation	Absent	32	84.20%	111	93.30%	13	86.70%	37	92.50%
	Present	6	15.80%	8	6.70%	2	13.30%	3	7.50%
Chronic inflammation	Absent	34	89.50%	108	90.80%	13	86.70%	30	75.00%
	Present	4	10.50%	11	9.20%	2	13.30%	10	25.00%
Other findings	Amniocyte Hyperplasia	5	21.70%	19	23.50%	0	0.00%	6	20.70%
	Chorangioma	0	0.00%	2	2.50%	1	11.10%	0	0.00%
	Chorangiomatosis	0	0.00%	2	2.50%	0	0.00%	1	3.40%
	Chorangiosis	9	39.10%	40	49.40%	6	66.70%	15	51.70%
	Delayed maturation	5	21.70%	14	17.30%	2	22.20%	4	13.80%
	Excessive fibrin	1	4.30%	0	0.00%	0	0.00%	0	0.00%
	Focal hydropic change	0	0.00%	1	1.20%	0	0.00%	0	0.00%
	Intervillous haematoma	3	13.00%	16	19.80%	0	0.00%	6	20.70%
	Laminar necrosis	1	4.30%	1	1.20%	0	0.00%	2	6.90%
	Peripheral infarct	0	0.00%	8	9.90%	2	22.20%	1	3.40%
	Pigmented macrophages	1	4.30%	6	7.40%	0	0.00%	2	6.90%
	Retroplacental haematoma	1	4.30%	6	7.40%	0	0.00%	0	0.00%
	Subchorionic haematoma	4	17.40%	6	7.40%	0	0.00%	4	13.80%

## Discussion

COVID-19 has proven to be a dynamic disease characterized by disease heterogeneity and variability of pathologic findings. Given the placenta’s role as the maternal-fetal interface, examination in pregnancies complicated by maternal SARS-Cov-2 infection is critical, as it may reveal mechanisms underlying fetal compromise. Histological evaluation can inform neonatal management, particularly in the NICU setting. Identification of placental malperfusion lesions may help exclude infectious etiologies and reduce unnecessary prolonged antibiotic use (11). Similarly, vaso-occlusive lesions consistent with FVM may warrant thorough neurological evaluation and supportive management to mitigate adverse sequelae such as stroke (11).

The evaluation showed that out of 234 placentas, 14.1% (33/234) showed features consistent with FVM, while 89.5% (201/234) did not. Cases with a history of a positive test for SARS-CoV-2 during pregnancy accounted for 45.5% (15/33) compared to 29.4% (59/201) of controls. Fisher’s exact test to evaluate for an association between SARS-CoV-2 infection and FVM in pregnancy yielded a *p*-value of 0.069 (Odds ratio: 2.01; Confidence interval: 0.95–4.24). Although tending towards statistical significance, this value did not reach the threshold p-value of <0.05. However, based on power calculation, the present study may have been underpowered to detect an effect between exposure to SARS-CoV-2 during pregnancy and FVM.

A study by Gulersen et al. (40) evaluated the histopathological features of 50 placentas in the setting of SARS-CoV-2 infection during pregnancy and compared them to historical controls. However, as was the case with the present study, they found no statistically significant difference in the prevalence of FVM between the two groups (8% in placentas from COVID-19-positive women versus 12% in historical controls, *p* = 0.74). There was also no observed statistical difference in other examined features, such as MVM, inflammation and chorangiosis. Similarly, a meta-analysis conducted by Suhren et al. (41) reviewed 30 publications to determine whether there would be any identifiable unique histological pattern associated with maternal COVID-19. Morphologic features of 1,452 placentas associated with COVID-19 maternal infection were reviewed. No specific pattern unique to COVID-19 was identified.

Macroscopic placental morphology did not appear to be significantly affected by SARS-CoV-2 infection. Most placentas in this study showed normal macroscopic features characterized by tan-brown to beefy-red parenchyma (Figure 2A). The most common abnormality was peripheral infarction (Figure 2D), observed in 22.7%(10/44) of placentas. These findings are comparable to those reported in a study of 50 placentas (42), of which 60% were unremarkable on gross examination and 40% demonstrated infarcts. The mean placental weight did not differ significantly between cases and controls (484.5 g vs. 481.1 g) with both values falling within normal limits. Umbilical cord insertion was predominantly eccentric in both cohorts (72.7% vs. 66.7%). Although marginal cord insertion has been reported to be more frequent in placentas from SARS-CoV-2 exposed women (43), no association has observed between maternal COVID-19 during pregnancy and cord insertion patterns. One placenta in this study associated with a COVID-19-positive mother showed gross, diffuse parenchymal abnormality characterized by solid tan-white foci with interspersed hemorrhagic areas (Figure 2B).

The macroscopic appearance may be partly attributable to COVID-19 infection, as there were no identifiable risk factors other than intrauterine fetal growth restriction. However, since this was the only placenta that exhibited evidence of the parenchymal viral presence and grossly recognizable features, the association between gross appearance and COVID-19 cannot be conclusively established. On microscopy, this placenta demonstrated extensive infarction and fibrin deposition (Figure 5D). Viral presence was detectable via PCR, which was conducted by the parent study in both the placenta and the neonate. This denoted the possibility of vertical transmission. These findings are similar to those of Valdespino-Vázquez et al. (44), who documented findings of an infarcted placenta, diffuse perivillous fibrin deposition and, additionally, inflammatory features characterized by active chronic intervillositis and subchorial inflammation in a case with vertical transmission. Kirstman et al. (45) also reported extensive infarction and chronic intervillositis attended predominantly by histiocytes in a mother whose neonate tested positive for COVID-19.

The findings in this study and those reported in the literature would argue that morphologic clues that would imply the possibility of a transplacental transmission would be the extent of placental injury, seen both grossly and/or microscopically and the presence of chronic histiocyte-predominant inflammation and trophoblast necrosis. This should prompt additional investigation with IHC or ISH to establish a diagnosis of placentitis with a possible risk of vertical transmission and recommended neonatal correlation. The possibility of vertical transmission is associated with neonatal outcomes, including but not limited to pneumonia (46, 53–55). Follow-up of these neonates is warranted to mitigate any long-term effects they may incur as a sequela of viral infection (47). The most frequent finding identified in women with COVID-19 during pregnancy in this study was chorangiosis (48.21%, 27/74). A similar observation has been reported in a study of SARS-CoV-2 exposed placentas, which also noted and increased frequency of chorangiosis (42). However, in contrast to our findings, most studies describe MVM, FVM or inflammatory lesions as the predominant histopathological features in placentas collected from women with SARS-CoV-2 infection (7, 10, 29, 36, 48, 49). Diagnosis of chorangiosis in this study was based on Altshuler’s criteria.

Chorangiosis has been shown to be associated with unfavorable neonatal outcomes and is regarded as a marker of chronic placental hypoxia (50). In this cohort, it was also observed in women without evidence of SARS-CoV-2 exposure (39.1%, *n* = 9), suggesting that other factors such as maternal comorbidities, subclinical hypoxia, placental adaptive responses may contribute to its development. The predominance of chorangiosis in this study could reflect subtle hypoxic stress during pregnancy, potentially amplified by SARS-CoV-2 infection. However, no statistically significant difference was observed between PCR/antigen-positive and PCR/antigen-negative participants.

This study included placentas from participants who exhibited a non-acute status at enrollment; PCR/antigen negative but positive for Immunoglobulin G (IgG) antibodies unrelated to vaccination. The presence of antibodies implies a prior exposure to COVID-19, detectable within a week of infection (51), but it does not inform the exact timing of infection. The existence of antibodies ranges from 1 to 2 years post-infection (16). While little can be accurately gleaned from this cohort of participants, it is interesting to note that they exhibit an almost similar frequency of microscopic features to participants with a proven active infection. This brings to the forefront the objective explored by Corbetta-Rastelli et al. (52) of whether the timing and severity of infection would affect outcomes. Their study examined 138 placentas collected from women who were COVID-19-positive before and after 20 weeks of gestation. While no specific histological signature was identified, they found that placental hallmarks associated with infection response (not defined in the study) were determined more frequently in women with COVID-19 infection before 20 weeks. No difference was noted in the occurrence of FVM, MVM or inflammation (52). This avenue would benefit from further exploration to address not only whether infection prior to pregnancy, early in pregnancy or late in pregnancy would have any differences in placental pathology but also whether antibodies in the mother would confer an immune advantage to the fetus.

## Conclusion

In this case–control study, maternal SARS-CoV-2 infection during pregnancy was not significantly associated with FVM in placentas. However, chorangiosis was frequently observed, suggesting a possible pattern of hypoxia-related placental changes that warrants further investigation in future studies. These findings contribute to the growing body of literature characterizing placental histopathology in SARS-CoV-2-exposed pregnancies and highlight the importance of comprehensive assessment.

## Strengths and limitations

Strengths include a rigorous and standardized histopathological evaluation and detailed characterization of placental lesions. However, there are a few limitations. Firstly, testing for SARS-CoV-2 was not trimester specific, limiting evaluation of the effect of timing of infection on the occurrence of any of the lesions described. Secondly, recruitment of participants beyond the study period was limited. A census sampling approach was adopted to mitigate this, and while this study shows the possibility and range of lesions that can occur in the event of infection, it was, however, underpowered.

## Data Availability

The original contributions presented in the study are included in the article/supplementary material, further inquiries can be directed to the corresponding author.
